# Effects of Different Degrees of Gelatinization on Structural, Physicochemical and Digestive Properties of Kudzu Starch

**DOI:** 10.3390/foods14213614

**Published:** 2025-10-23

**Authors:** Zirui He, Fan Zhu, Mei Li, Xiangli Kong

**Affiliations:** 1 Key Laboratory of Nuclear Agricultural Sciences of Ministry of Agriculture and Rural Affairs, Institute of Nuclear Agricultural Sciences, College of Agriculture and Biotechnology, Zhejiang University, Hangzhou 310058, China; zirui.h@outlook.com; 2 School of Chemical Sciences, The University of Auckland, Private Bag 92019, Auckland 1142, New Zealand; fzhu5@yahoo.com; 3 Analysis Center of Agrobiology and Environmental Sciences, Zhejiang University, Hangzhou 310058, China; limei7251314@zju.edu.cn

**Keywords:** kudzu starch, degree of gelatinization, structural characteristics, functional properties, *in vitro* digestion

## Abstract

Kudzu (*Pueraria* spp.) starch, valued for its transparency, viscosity, and stability, has broad potential in functional and instant food applications. However, its limited cold-water solubility and inconsistent functional performance across cultivars hinder wider utilization. To improve its processability and nutritional functionality, this study aimed to elucidate how the degree of gelatinization (DG)—a structural indicator of starch transformation—can be precisely controlled and used to modulate starch properties. Starches from two typical kudzu cultivars, K10 (*Pueraria thomsonii*) and K27 (*Pueraria lobata*), were subjected to hydrothermal treatment (45–95 °C) to obtain samples with defined DG levels. DG was quantitatively determined by enzymatic assay, differential scanning calorimetry (DSC), and iodine-binding analysis, enabling method cross-validation. Increasing DG enhanced iodine complexation capacity, elevated gelatinization temperatures, and reduced enthalpy change and crystallinity. K27 exhibited more pronounced physicochemical transitions at lower DG than K10, indicating cultivar-specific sensitivity. In vitro digestion revealed that hydrolysis kinetics gradually approached and eventually conformed to a first-order model as DG increased, confirming a DG-dependent shift in digestibility. These results establish DG—rather than processing temperature—as the primary factor governing kudzu starch functionality and provide a methodological basis for designing starch-based foods with tailored glycemic and textural properties.

## 1. Introduction

Kudzu (*Pueraria* spp.), a perennial vine of the Fabaceae family, is primarily classified into *Pueraria thomsonii* and *Pueraria lobata* [1], and is widely cultivated in East Asia for both food and medicinal use [2]. Beyond its bioactive compounds, kudzu is rich in starch, which accounts for approximately 52% (*w*/*w*) of its dry weight and has a relatively high amylose content (average 24.76%) [1,3]. These features confer distinctive physicochemical properties—such as high paste transparency, strong viscosity, and stability—that support its application in instant foods, jellies, and candies [4,5]. However, poor cold-water solubility and nutrient loss during extraction still limit its broader utilization. Since starch characteristics vary markedly between kudzu cultivars [1], comparative studies can provide insights for improving both processing adaptability and product performance. In particular, K10 (derived from *Pueraria thomsonii*) and K27 (derived from *Pueraria lobata*) differ in granule morphology, amylose content and gelatinization temperatures, suggesting potential functional divergence that remains insufficiently understood.

Gelatinization, the heat- and moisture-induced transformation of starch granules, reduces crystallinity and alters rheological behavior, affecting texture, digestibility, and shelf life [6,7]. The degree of gelatinization (DG) is a critical determinant of starch functionality, influencing rheological properties [8], lubrication [9], glycemic response [10], and overall quality [11]. Although temperature governs the onset of gelatinization, DG reflects the intrinsic structural disassembly of starch granules and therefore can be treated as an independent structural variable rather than a passive consequence of heating. This distinction allows for isolating structural effects from processing parameters when evaluating starch functionality. Accurately quantifying this critical parameter, however, is method-dependent. Common techniques—enzymatic, iodine-binding, and differential scanning calorimetry (DSC)—probe different aspects of gelatinization, from molecular accessibility and amylose leaching to thermal energy absorption, each with its own scope and limitations [12]. Therefore, employing a multi-method approach is essential for a comprehensive and reliable assessment of DG, enabling the decoupling of its effects from the mere influence of processing temperature.

Recent studies have further emphasized the influence of botanical origin and granular architecture on starch gelatinization across cereals, tubers, and legumes [11,12,13], underscoring the need to investigate less-studied sources such as kudzu starch to elucidate their unique structure–function relationships. However, despite growing interest, there remains a lack of systematic comparison between kudzu cultivars under controlled DG gradients, and the methodological consistency in DG determination has rarely been addressed. Clarifying these aspects is crucial for identifying cultivar-specific behaviors and optimizing starch modification strategies for tailored food applications [14,15,16].

In this study, starches from two kudzu cultivars (*Pueraria thomsonii*, K10; *Pueraria lobata*, K27) were hydrothermally treated to achieve defined DG levels. DG was determined using three independent methods—enzymatic assay, DSC, and iodine-binding—to enable cross-validation and method comparison [12]. The effects of DG on physicochemical, structural, and digestive properties were analyzed to decouple DG influences from processing temperature. The findings establish DG as a key driver of functional transitions in kudzu starch, providing a basis for tailoring starch-based foods with specific health and sensory attributes.

## 2. Materials and Methods

### 2.1. Materials

Two typical cultivars of kudzu used in the experiment were from China, with the following varieties and origins: K10 (*Pueraria thomsonii*, Teng County, Wuzhou City, China) and K27 (*Pueraria lobate*, Gejiu City, China). α-Amylase from porcine pancreas (≥5 units/mg, CAS: 9000-90-2) was obtained from Sigma-Aldrich Co., Ltd. (St Louis, MO, USA). Amyloglucosidase from Aspergillus niger (100,000 units/mL, CAS: 9032-08-0) was sourced from Aladdin Chemistry Co., Ltd. (Shanghai, China). The D-Glucose Assay (GOPOD) kit was purchased from Megazyme (Bray, Ireland). All reagents used were of analytical grade.

### 2.2. Preparation of Kudzu Starch Samples with Different DG

Kudzu starch was extracted following the procedure of a previous study [17], with some modifications. The kudzu roots were cleaned and peeled. The middle, stout segments of the roots were selected, cut, and crushed. The resulting kudzu grout was then sieved successively through 100, 270, and 400-mesh sieves. The mixture was centrifuged at 3000× *g* for 10 min; the supernatant and upper impurities were discarded, while the white precipitate at the bottom was retained. The precipitate was repeatedly washed with Milli-Q water by centrifugation until a homogeneous white pellet was obtained. The final washed precipitate was spread on a flat dish, dried at 40 °C for 24 h, and passed through a 100-mesh sieve to obtain two varieties of kudzu starch.

Samples of kudzu starch with different DG were prepared by a controlled heating method in aqueous suspension. Two grams of prepared kudzu starch were mixed with 20 mL of Milli-Q water and heated at 45 °C, 55 °C, 65 °C, 75 °C, 85 °C, and 95 °C (below the boiling point of water, 100 °C) for 30 min, with continuous agitation. The resulting starch solution was then centrifuged at 3000× *g* for 10 min. The supernatant was discarded, and the precipitate was washed several times with Milli-Q water. After freeze-drying, the starch was passed through a 100-mesh sieve. All samples were stored at 4 °C until use.

### 2.3. Particle Size Distribution

The particle size distribution of the samples was characterized using a Mastersizer 3000 (Malvern Instruments Ltd., Malvern, UK). Starch sample (0.1 g) was weighed and mixed in 10 mL of Milli-Q water. The starch suspension was then added drop by drop into the sample cell. The ultrasonic dispersion device was conducted to uniformly disperse the sample for 1 min. The particle size measurement was performed, and the data were automatically processed by the associated computer software. The parameters used were as follows: shading intensity of 10−20%, refractive index of the sample particles of 1.54, particle absorption rate of 0.01, analytical mode universal, dispersant Milli-Q water (refractive index 1.33), and the following eigenvalues were recorded: D_10_, D_50_, D_90_, and D_[4,3]_. Additionally, the Span value, which represents the distribution span of the sample, was calculated:

(1) $$\mathrm{Span}\;=\;\frac{D_{90}-D_{10}}{D_{50}}$$

where D_10_ is the 10th percentile particle size (µm), D_50_ is the median particle size (µm), D_90_ is the 90th percentile particle size (µm), and Span is the particle size distribution span.

### 2.4. Apparent Amylose Content

The apparent amylose content (AAC) was determined according to our pervious method with some modifications [18]. Anhydrous ethanol (100 μL) and 900 μL of 1 M NaOH solution were added to 10 mg of starch sample (dry weight). The suspension was thoroughly mixed by vortexing, and then boiled in a water bath for 15 min. After cooling, the supernatant was collected and diluted to a final volume of 10 mL with Milli-Q water. A 200 μL aliquot of the resulting solution was mixed with 3.8 mL of iodine indicator (a mixed solution of I_2_-KI-acetic acid). Absorbance values were recorded at 620 nm after 10 min using a UV spectrophotometer (SP-756P, Spectrum Instruments, Shanghai, China). The AAC of the samples was calculated based on standard curves established with different ratios of amylose and amylopectin blends.

### 2.5. Amylose Leaching

Amylose leaching (AML) was analyzed according to a previous study with some modifications [19]. Kudzu starch (20 mg, dry basis) was dispersed in 10 mL water and heated at 55 °C, 65 °C, 75 °C, 85 °C and 95 °C for 30 min, respectively, with shaking every minute to maintain the suspension. The tubes were then cooled to room temperature and centrifuged at 3000× *g* for 10 min. The supernatant (1 mL) was withdrawn, and the amylose content was determined as described in Section 2.4. AML was expressed as the percentage of amylose leached from starch.

### 2.6. Digestive Properties

The digestive properties of starch were analyzed using in vitro enzyme digestion experiments.

#### 2.6.1. Digestive Properties of Raw Starch

A total of 50 milligrams of raw starch was dispersed in 4 mL of sodium acetate buffer (0.5 M, pH 5.2, 5 mM CaCl_2_), heated in a boiling water bath for 30 min, cooled and kept at 37 °C. Enzyme mixture (1 mL, 36 U/mL, α-amylase; 18 U/mL, amyloglucosidase) was added, and the mixture was shaken at 37 °C for 2 h. At the time points of 0, 20, 30, 60, 90 and 120 min, 200 μL of the sample was withdrawn, and 800 μL of anhydrous ethanol was added to inactivate the enzyme [20]. The supernatant was then centrifuged at 10,000× *g* for 10 min, and the glucose content of the supernatant was quantified using GOPOD assay kit to calculate the starch composition, hydrolysis rate, and plot the digestion curve.

The contents of rapidly digestible starch (RDS), slowly digestible starch (SDS) and resistant starch (RS) were calculated using the following equations [21]:

RDS = 0.9 × (G_20_ − G_0_) (2)

SDS = 0.9 × (G_120_ − G_20_) (3)

RS = TS − RDS − SDS(4)

where G_0_, G_20_ and G_120_ represent the mass fraction of glucose produced by hydrolysis at 0, 20, and 120 min, respectively (%). TS represents the mass fraction of total starch in the sample (%), and 0.9 is the conversion factor.

The rate of starch hydrolysis (S*_H_*) is calculated as follows [22]:

(5) $$S_{H}\;=\;0.9\;\times \;\;\frac{G_{p}}{S_{i}}$$

where *S_H_* is the percentage of starch hydrolyzed (%), *G_p_* is the glucose yield (g), and *S_i_* is the initial amount of starch (g).

The digestion curve conforms to the first-order kinetic equation, and the area under the hydrolysis curve (AUC), hydrolysis index (HI), and estimated glycemic index (eGI) are calculated by the following equations [23]:

(6) $$C=C_{\infty }\left(1-e^{-kt}\right)$$

(7) $$AUC=C_{\infty }\left[(t_{f}-t_{0})-\frac{1-e^{-k\left(t_{f}-t_{0}\right)}}{k}\right]$$

(8) $$\mathrm{HI}=\frac{{AUC}_{sample}}{{AUC}_{white\;bread}}$$

eGI = 39.71 + 0.549 × HI(9)

*C*: the percentage of hydrolyzed starch at digestion time t (min);

*C*_∞_: the percentage of hydrolyzed starch at 120 min;

*k*: the kinetic constant (min^−1^);

*t_f_*: 120 min;

*t*_0_: 0 min;

AUC: the area under the starch hydrolysis curve;

HI: the hydrolysis index, which indicates the relationship between the sample and the reference food (white bread).

#### 2.6.2. Digestive Properties of Kudzu Starch with Different DG

Partially gelatinized starch (50 mg) was dispersed in 4 mL of sodium acetate buffer (0.5 M, pH 5.2, 5 mM CaCl_2_) and held at 37 °C. The enzymatic reaction was then carried out as described in Section 2.6.1. The hydrolysis rate of the samples was calculated, and the digestion curve was plotted.

### 2.7. Thermal Properties

Thermal properties were analyzed using a differential scanning calorimeter (DSC) (Q100, TA instruments, New Castle, DE, USA). Starch (2 mg, dry basis) was weighed into an aluminum crucible, and 6 μL of Milli-Q water was added. The sealed crucible was equilibrated overnight at room temperature to balance the moisture, and then heated from 40 °C to 130 °C at a rate of 10 °C/min. An empty pan was used as the reference. Thermal parameters including *T*_o_ (onset temperature), *T*_p_ (peak temperature), *T*_c_ (conclusion temperature), and ∆*H* (gelatinization enthalpy change) were calculated using Universal Analysis Program, version 4.4A.

### 2.8. Iodine-Binding Capacity (IBC)

The method described by a previous report was followed with slight modifications [24]. Raw starch (10 mg) and 1 mL Milli-Q water were mixed evenly, heated in a water bath for 30 min with different temperature (45 °C, 55 °C, 65 °C, 75 °C, 85 °C, 95 °C), diluted to 1 mg/mL suspension and cooled to room temperature for measurement. Iodine solution (800 μL) was mixed with 200 μL of the starch suspension to be tested (with 200 μL Milli-Q water as the blank control group). The absorbance was recorded from 500 nm to 900 nm using a UV spectrophotometer (SP-756 P, Spectrum Instruments, Shanghai, China) with a step size of 2 nm, and the scanning spectrum was plotted.

### 2.9. Determination of DG

The DG of starch was measured using three methods, with appropriate modifications [12].

#### 2.9.1. Enzymatic Method

The method followed the procedure outlined in Section 2.6, and DG was calculated using the following formula:

(10) $$\mathrm{DG}\%\;=\;\frac{A_{120}^{\prime }}{A_{120}}\times 100\%$$

where A′_120_ is the absorbance after 120 min of enzymatic digestion of the partially gelatinized sample (Abs); A_120_ is the absorbance after 120 min of enzymatic digestion of the corresponding raw starch (Abs).

#### 2.9.2. DSC Method

The method followed the procedure outlined in Section 2.7, and DG was calculated using the following formula:

(11) $$\mathrm{DG}\%\;=\;(1-\frac{{∆H}^{\prime }}{∆H})\times 100\%$$

where ∆*H*′ is the enthalpy change of samples with various DG (J/g), and ∆*H* is the enthalpy change of the corresponding raw starch (J/g).

#### 2.9.3. Iodine-Binding Method

The method followed the procedure outlined in Section 2.8, and DG was calculated using the following formula:

(12) $$\mathrm{DG}\%\;=\;\frac{A^{\prime }}{A}\times 100\%$$

where A′ is the absorbance value at 620 nm for samples with different DG (Abs), and A is the absorbance value at 620 nm of raw starch treated in boiling water bath (Abs).

### 2.10. Scanning Electron Microscope (SEM)

The morphology of the samples was observed using a scanning electron microscope (GeminiSEM 300, Carl Zeiss, Oberkochen, Germany). The samples were mounted on a carrier stage with conductive adhesive and subjected to vacuum gold plating. The morphology of the samples was then observed under an accelerating voltage of 3.0 kV [25].

### 2.11. X-Ray Diffraction (XRD)

An X-ray diffractometer (Bruker AXS, Karlsruhe, Germany) was used to analyze the long-range ordered structure and relative crystallinity (RC) of the samples. The test conditions were as follows: accelerating voltage of 40 kV, a current set to 100 mA, a step size of 0.04°, a scan rate of 1°/min, and a scanning range of 4−40° [26]. The RC of different samples was analyzed by Jade 6.5 software and calculated using the following equation:

(13) $$\mathrm{RC}\;=\;\frac{A_{c}}{A_{c}+A_{a}}\times 100\%$$

where A_c_ denotes the area of crystalline peaks on the X-ray pattern and A_a_ denotes the area of non-crystalline regions.

### 2.12. Fourier Transforms Infrared (FTIR) Spectroscopy

The short-range ordered structure of the samples was determined using Fourier Transform Infrared Spectroscopy (Thermo Fisher Scientific Co., Waltham, MA, USA), based on the method described by a previous study with slight modifications [27]. Sample (2 mg) was diluted 100 times with potassium bromide, thoroughly mixed, ground, and pressed for testing. The scanning range was 4000−500 cm^−1^ with a resolution of 4 cm^−1^ and 32 scans were performed using Milli-Q water as a blank [28].

### 2.13. Rheology

Rheological tests of the sample suspension (10%, *w*/*v*) were performed using a rheometer (DHR-1, TA Instruments, Waters, New Castle, DE, USA) with a parallel plate geometry (40 mm diameter, 1000 μm gap). Prior to each test, the samples were equilibrated at 25 °C for 30 s. A shear sweep test (0.01–100 s^−1^) was conducted to determine the relationship between the apparent viscosity of the sample and the shear rate. A power-law model was used to describe the flow behaviors of the starch gels [29]:

(14) $$\tau =K{\left(\gamma \right)}^{n}$$

where τ is the shear stress (Pa), γ is the shear rate, *K* is the consistency index (Pa·s^n^), and n is the flow behavior index.

### 2.14. Statistical Analysis

To ensure the accuracy and credibility of the experiment, all measurements were conducted with more than 3 independent replications for each group, and the results were expressed as means ± standard deviations. The data were analyzed by SPSS 26.0. The experimental data were analyzed using SPSS 26.0 (SPSS Inc., Chicago, IL, USA) with one-way analysis of variance (ANOVA) followed by Duncan’s multiple range test for post hoc comparisons at a significance level of 5% (*p* < 0.05). The data were then plotted using Origin 8.0 (OriginLab Corporation, Northampton, MA, USA).

## 3. Results and Discussion

### 3.1. Starch DG Determined by Three Different Methods and Their Correlations

#### 3.1.1. Enzymatic Method

The enzymatic method for determining DG is based on the assumption that there is a linear relationship between enzymatic sensitivity and DG [30]. Starch is hydrolyzed into reducing sugars using specific enzymes, such as amyloglucosidase [16], β-amylase–pullulanase [31], and β-amylase-isoamylase [32]. The concentration of reducing sugars is then determined using a chemical method, which allows for the calculation of the DG value.

The results from the enzymatic method showed that the DG increased gradually with the increase in temperature. The average DG for all kudzu starch samples treated at 45 °C was 0.87%, indicating that the starch did not begin to gelatinize, while the average DG at 95 °C was 99.25%, indicating nearly complete gelatinization. The DG values for different kudzu starches treated at 55 °C, 65 °C, 75 °C, and 85 °C treatments exhibited significant differences. As shown in the Figure S1a, the DG of the K27 was significantly higher than that of K10 under treatment of 55−75 °C, and the DG of K27 reached 74.14% after treatment at 75 °C. Increasing the temperature from 75 °C to 85 °C had little effect on DG, resulting in a “flat zone” with almost no increase. However, the DG increased significantly from 75.06% to 99.20%, achieving complete gelatinization when the temperature was raised from 85 °C to 95 °C. The appearance of the “flat zone” may be related to the conclusion temperature (*T*_c_) for this variety of starch (Table S2). And this was further supported by the fact that the DG of the K10 increased steadily as the temperature raised from 55 °C to 95 °C, with no “flat zone”.

#### 3.1.2. DSC Method

The principle of DSC is based on comparing the heat difference between a sample and a reference to detect various physical or chemical changes that occur in the sample at specific temperatures [33]. The enthalpy change (∆*H*) of starch represents the energy required to disrupt its double helix and crystal structure [34]. The DSC method determines the DG of starch by measuring ∆*H*.

The results determined by the DSC method showed a similar trend to the enzymatic method of 3.1.1, but its initial DG value was higher, probably due to the fact that the treatment at 45 °C had already slightly affected the starch granule [35], which changed the ∆*H*, and the substitution into the calculation formula led to a large result. Meanwhile, when the temperature was increased to 95 °C, the DSC curve was almost flat and ∆*H* could not be obtained to calculate DG, so there were some limitations. The increase in DG was more pronounced for K27 between 55 °C and 65 °C, while K10 exhibited a greater increase in DG between 65 °C and 85 °C (Figure S1b). This difference was mainly related to the onset temperatures (*T*_o_) of the two varieties (Table S2). To was lower than 65 °C for K27, and higher than 80 °C for K10. After the temperatures exceeded 95 °C, the starch approached or reached the state of complete gelatinization. The hydrogen bonds between molecules were largely broken, and the expansion of starch granules reached its maximum. As a result, the heat flow change became minimal and could not be measured accurately [36].

#### 3.1.3. Iodine-Bonding Method

The helical structure of amylose can bind to iodine to form a blue complex [37]. When starch is gelatinized, the amount of amylose that leaches out increases, leading to greater iodine-binding [38]. The iodine-binding method determines the DG by comparing the ability of the starch to form a blue complex with iodine.

The difference in DG versus temperature measured by the iodine-binding method between *Pueraria thomsonii* starch (K10) and *Pueraria lobate* starch (K27) was even more pronounced (Figure S1c). The DG values of K27 were significantly higher than those of K10 under the treatments of 55−85 °C. The heating from 75 °C to 85 °C greatly accelerated the increase in DG for K10. Finally, from 85 °C to 95 °C, the growth rate of DG in both varieties slowed significantly. This was mainly related to the leaching rate of amylose at the corresponding temperatures [39].

#### 3.1.4. Comparison of Three Methods for Determining Starch DG

**Figure 1:**
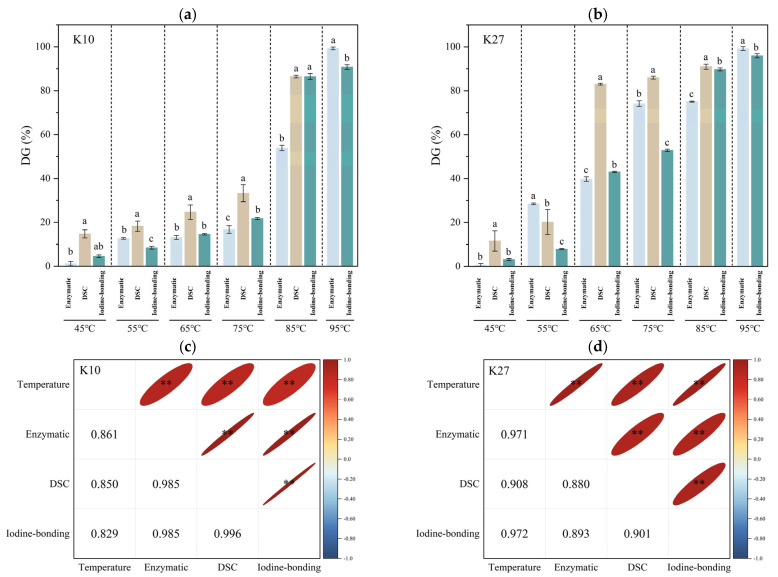
DG analysis of kudzu starches using three methods. (**a**) K10; (**b**) K27; (**c**) Temperature–DG correlation for K10; (**d**) Temperature–DG correlation for K27. Different lowercase letters indicate significant differences (*p* < 0.05); ** *p* < 0.01.

All three methods were able to reflect the trend of increasing DG of starch with rising treatment temperature at the initial stages (Figure 1). However, the standard deviation of DG determination using DSC was generally larger, which was consistent with the findings of Di Paola et al. [40]. This may be due to the fact that the partially gelatinized starch exhibited varying DG across different granules or within different parts of the same granule, resulting in differences in the onset, peak, and conclusion points of the gelatinization reaction during each determination. These variations can affect the stability and reproducibility of the ∆*H* [38]. Importantly, significant differences (*p* < 0.05) among the three methods were observed across different treatment temperatures (Figure 1). This indicates that the choice of an optimal method is context-dependent and should be aligned with specific experimental conditions. The distinct characteristics of the three methods, which help explain these observed differences, are summarized in Table S1.

For *Pueraria thomsonii* starch (K10), the values obtained by the DSC method were markedly higher than those determined by the enzymatic and iodine-binding methods at 45 °C, 55 °C, 65 °C, and 75 °C. This observation, considering the standard deviations, indicates a distinct trend where DSC overestimates DG in the low-to-intermediate temperature range for this variety. This discrepancy may be due to the fact that ∆*H* was highly sensitive to temperature variations [34,38], and it is affected and reduced even when gelatinization had almost not started, as observed in this study at 45 °C. This observation, combined with the principles outlined in Table S1, suggests that the three methods probe different structural aspects during gelatinization: the DSC method detects the early disruption of the crystalline structure (reflected by ∆*H*), while the enzymatic and iodine-binding methods are dependent on the subsequent physical disintegration of granules and the leaching of amylose, respectively [12]. The higher *T*_o_ of K10 (80.36 °C, Table S2) indicates a more stable crystalline structure, whose initial disruption is sensitively captured by DSC ahead of the changes detected by the other two methods. Therefore, the enzymatic and iodine-binding methods were more suitable to obtain DG for temperatures below 75 °C.

For *Pueraria lobate* starch (K27), the DSC method showed a steep increase in DG with elevated temperatures from 45 °C to 55 °C, after which the rate of increase slowed. This behavior aligns with its lower *T*_o_ (61.72 °C, Table S2), suggesting the rapid melting of less thermally stable crystalline regions at lower temperatures. In contrast, the enzymatic and iodine-binding methods provided a more comprehensive view of the gradual increase in DG as the hydrothermal treatment temperature rose.

#### 3.1.5. Correlation of Three Methods for Determination of Starch DG

As shown in Figure S1, which includes the standard deviation bars and labels for significant differences, the DG values obtained for the two kudzu starch varieties differed significantly depending on the method used. This strongly suggests that the optimal method for DG determination is starch-source-dependent. Therefore, Pearson’s correlation analysis was performed to examine the relationships among the three methods and with the treatment temperature, in order to identify the most suitable method for each variety.

For *Pueraria thomsonii* starch (K10), the results from the enzymatic method showed a significant positive correlation with both the iodine-binding method and the DSC method (*p* < 0.01), with the correlation coefficients of 0.985 for all three methods, indicating strong agreement between them (Figure 1c). Furthermore, the correlation coefficients between the treatment temperature and the determination methods were as follows: enzymatic > DSC (except at 95 °C) > iodine-binding method. This suggested that the enzymatic method was more sensitive to temperature variations and could more effectively reflect the effects of different treatment temperatures on the DG of *Pueraria thomsonii* starch. Therefore, the enzymatic method is the most suitable choice for determining the DG of *Pueraria thomsonii* starch.

For *Pueraria lobate* starch (K27), the results from the iodine-binding method also showed a significant positive correlation (*p* < 0.01) with both the enzymatic method and DSC method, and their correlation coefficients were 0.893 and 0.901, respectively (Figure 1d). This indicated a strong agreement among the three methods. Additionally, the correlation coefficients between the treatment temperature and the determination methods were as follows: iodine-binding method > enzymatic method > DSC method (except at 95 °C). Therefore, for *Pueraria lobate* starch, iodine-binding method was the most suitable assay among the three.

### 3.2. In Vitro Digestive Properties

#### 3.2.1. Digestive Properties of Raw Starch

The digestive properties of starch are influenced by its botanical source, variety, and multi-scale structure, including amylose content, granule morphology, and lamellar organization, among other factors that affect its digestibility [41,42,43]. Starch can be classified into three different fractions based on differences in the rates of its hydrolysis and absorption in the digestive tract: rapidly digestible starch (RDS), slowly digestible starch (SDS), and resistant starch (RS). In addition, first-order kinetic modeling is an effective approach for describing starch digestion kinetics and quantitatively comparing the rate and extent of starch digestion [44]. The digestion parameters of raw kudzu starch are shown in Table S2.

We compared the starch composition of kudzu starch with common starches [45,46,47]. The RDS contents of K10 and K27 were 78.21% and 81.18%, respectively, which were similar to those of rice starch and significantly higher than those of maize starch and potato starch. The SDS content was 7.38% and 12.69%, respectively, similar to that of maize starch, slightly higher than that of potato starch, and much higher than that of rice starch. The RS content was 14.41% and 6.13%, higher than that of maize starch and rice starch. Overall, kudzu starch exhibited a high RDS content, indicating a capacity for rapid energy supply; the SDS content was moderate, providing a sustained energy supply; and a high RS content was associated with various health benefits, including the regulation of blood glucose levels, enhancement of gut health, defense against pathogens, and the deliberate establishment and maintenance of lifestyle practices conducive to achieving and sustaining an ideal body weight [48,49]. Kudzu starch’s balanced RDS, SDS, and RS composition can provide quick and sustained energy while supporting health benefits.

Among varieties, *Pueraria thomsonii* starch (K10) exhibited higher RS content and lower RDS content compared to *Pueraria lobate* starch (K27), which may be related to variety-specific genetic characteristics and growth environment [50]. These differences in starch composition could influence the texture and digestibility of food products made from these varieties. *Pueraria thomsonii*, with its higher RS content, might offer more health benefits, such as aiding in weight management and gut health. On the other hand, *Pueraria lobate*’s lower RS and higher RDS content could result in a faster energy release, potentially impacting the glycemic response [51].

The digestive differences between K10 and K27 can be further elucidated by their structural parameters (Table S2). K10’s higher RS and lower RDS align with its significantly higher amylose content (27.69% for K10 compared to 26.51% for K27), as a higher amylose content is a key factor in forming enzyme-resistant structures [43], and its smaller granule size (e.g., D_50_: 8.76 μm for K10 versus 9.55 μm for K27), which may restrict enzyme accessibility [45]. It is noteworthy that despite K27’s higher initial crystallinity (RC: 17.15% for K27 in contrast to 15.36% for K10, Table S3), it exhibited a lower RS content. This observation suggests that, for kudzu starch, amylose content and granule size could be more decisive factors than crystallinity in governing resistant starch formation and overall digestibility.

The average glycemic index (GI) of kudzu starch was 43.36, with little variation between the two varieties. Therefore, kudzu starch can be classified as a low-GI food [52], characterized by a long residence time in the gastrointestinal tract, low absorption rate, slow glucose release, low peak glucose levels in the bloodstream, gradual decline in blood glucose, and minimal impact on blood glucose levels. The low-GI property of kudzu starch aids in controlling postprandial blood glucose levels while promoting satiety, which contributes to effective weight management.

#### 3.2.2. Effects of Different DG on the Digestive Properties of Kudzu Starch

**Figure 2:**
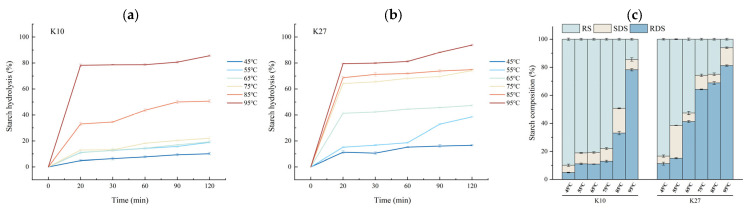
In vitro digestion of kudzu starches with different DG. (**a**) Hydrolysis curves of K10; (**b**) Hydrolysis curves of K27; (**c**) Nutritional composition.

The hydrolysis rate of all samples increased with the rise in treatment temperature, although the rate of increase varied between the two kudzu starch samples (Figure 2). When the treatment temperature reached 95 °C, all samples exhibited higher starch hydrolysis rates during the first 60 min, after which the rates began to plateau. This pattern was consistent with the first-order kinetic model [53]. However, when the treatment temperature was lower than 95 °C, the digestion curves differed with the treatment temperature and did not align with the first-order kinetic model. Specifically, when the treatment temperature was 45 °C, the digestion curve remained flat, with the final hydrolysis rate of each sample being lower than 20%. When the treatment temperature was 55 °C, the digestion curve of K10 still remained flat, while the curve for K27 showed a slope, with the hydrolysis rate continuing to increase as digestion time was prolonged. The hydrolysis rate significantly increased between 60 and 120 min. As the temperature continued to rise, the digestion curves of all samples began to show a noticeable slope. Finally, at 95 °C, the digestion curves for all kudzu starch varieties conformed to the first-order kinetic model.

Overall, as the DG of kudzu starch increased from 0% to 100%, the digestion profile transitioned from a relatively flat curve to a stepwise ascent, ultimately conforming to a first-order kinetic model. These findings are critical for understanding and predicting the digestive behavior of kudzu starch under various processing and cooking conditions.

The starch nutritional composition of kudzu starch with different DGs is shown (Figure 2c). The RDS of kudzu starch increased, while the RS decreased as the treatment temperature increased from 45 °C to 95 °C. This suggested a close relationship between starch structure and digestibility [54]. Ungelatinized starch granules exhibit densely packed crystalline lamellae, which limit amylase access and make digestion slow. With the increase in treatment temperature, kudzu starch granules underwent a transition from ordered to disordered structure, a process in which starch granules absorbed water and swelled, with amylose gradually dissolving to form a colloidal solution [55]. As the DG increases, the starch crystallites are broken down, leading to a loss of molecular order within the granules and a transition from an orderly to a disordered state. This disruption increases the accessibility of the starch to amylase, thereby accelerating starch hydrolysis [56]. Since the contents of RDS and RS were calculated based on the hydrolysis rate, RDS refers to the amount of starch that can be enzymatically hydrolyzed within 20 min under simulated digestion conditions, whereas RS is the amount of starch that cannot be enzymatically hydrolyzed under the same conditions. Therefore, as the DG value increased, the kudzu starch was more easily enzymatically hydrolyzed and the calculated RDS increased while the RS decreased.

In addition, fluctuations in SDS content were observed during this period, which might be related to the granular structure of starch, the ratio of amylose to amylopectin, and the physical state of the starch [41]. Starch with a higher content of amylose is more likely to form SDS and RS after gelation, while starch with higher content of amylopectin is more likely to form RDS [57]. It is noteworthy that the treatment temperature corresponding to the group with the highest SDS content coincided with the point where the digestion curve transitioned from not conforming to the first-order kinetic model to conforming to it. This observation further validated the results of the digestion curve analysis.

### 3.3. Effects of Different DG on the Thermal Properties of Kudzu Starch

*T*_o_, *T*_p_, and *T*_c_ of the two kudzu starch samples increased with increasing treatment temperatures, while ∆*H* decreased continuously (Table 1). This suggests that the thermal stability of starch increased after partial gelatinization, requiring higher temperatures to complete the gelatinization process. Gelatinization involves the structural transformation of starch granules under hydrothermal conditions, including water absorption, swelling, and the disintegration of molecular structures [34]. The observed increase in *T*_o_, *T*_p_, and *T*_c_ aligns with trends commonly observed in annealing treatments [58], likely resulting from enhanced molecular interactions within starch granules, such as strengthened hydrogen bonding, which require more energy to disrupt [59]. High-temperature treatment may have also promoted the reorganization of amylose and amylopectin, altering pasting characteristics and necessitating higher gelatinization temperatures [60]. However, unlike annealing, which typically leads to an increase in ∆*H* due to improved crystallinity [58], the observed decrease in ∆*H* in this study indicates a different phenomenon. This reduction may be due to partial gelatinization, which caused changes in the crystalline structure of starch granules. These structural modifications likely reduced the disruption of intermolecular interactions during gelatinization, thereby the starch requiring less energy to complete the process [61].

### 3.4. Effects of Different DG on the Iodine-Binding Capacity of Kudzu Starch

**Figure 3:**
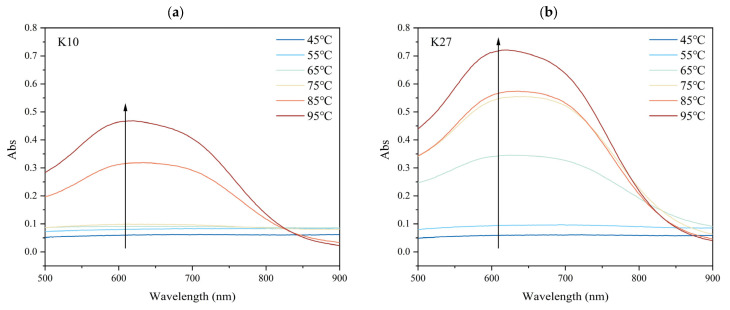
Iodine-binding curves of kudzu starches with different DG. (**a**) K10; (**b**) K27. The arrows indicate an increase in absorbance peak intensity.

The iodine-binding capacity of starch is related to its degree of polymerization and branching pattern [62]. The iodine-binding curve of kudzu starch that did not undergo gelatinization (treated at 45 °C) was almost completely flat in the wavelength range of 500−900 nm, with no peaks, and no significant differences were observed between different sources of kudzu starch (Figure 3). Upon heating from 45 °C to 55 °C, the curve remained flat, but the absorbance values of the various samples slightly increased. After warming from 55 °C to 65 °C, K27 showed noticeable changes: the absorbance value increased significantly and peaked at near 620 nm, while the curves of other kudzu starch samples remained flat. As the treatment temperature increased further, the absorbance values of each sample began to rise, and a curve appeared with a peak absorbance near 620 nm. This trend became more significant with even higher temperature. Meanwhile, comparing the changes in kudzu starch of the two varieties. Under low-temperature treatments (45 °C and 55 °C), the starch DG was small, and no significant difference in absorbance was observed between the K10 and K27. However, with further increases in temperature, the increase in absorbance for K27 was significantly higher than that of K10.

When amylose interacts with iodine, they form a blue complex with a spiral structure, exhibiting a maximum absorption wavelength (λ_max_) in the range of 600−640 nm. While amylopectin interacts with iodine to form a purplish-red complex, with λ_max_ in the range of 520−560 nm [63]. Based on the experimental results, the maximum absorption peaks of different kudzu starch samples were observed within the range of 600−640 nm, indicating the formation of amylose-iodine complexes.

During starch gelatinization, the structure of starch granules undergoes significant changes as the granules swell. The amylose chains separate from the granules and leach into the surrounding water, where they combine with iodine molecules to form the blue complexes [64]. As the treatment temperature increased, the DG of the kudzu starch also increased, leading to greater amylose release. This, in turn, enhanced the starch’s ability to bind with iodine, resulting in increased absorbance.

Furthermore, a quantitative correlation was observed between amylose release (reflected by iodine-binding capacity) and the degree of gelatinization (DG) obtained from DSC measurements. As shown in Section 3.1.4, the DG values determined by the iodine-binding method and DSC exhibited a highly significant positive correlation (*p* < 0.01) for both K10 and K27. This indicates that the increase in iodine absorbance quantitatively mirrors the progressive disruption of crystalline regions and the concurrent leaching of amylose detected by DSC [34,38]. Therefore, the iodine-binding method can serve as a reliable and sensitive indicator of starch gelatinization, complementing the thermal transition data from DSC [6,16].

### 3.5. Effects of Different DG on the Morphology of Kudzu Starch

**Figure 4:**
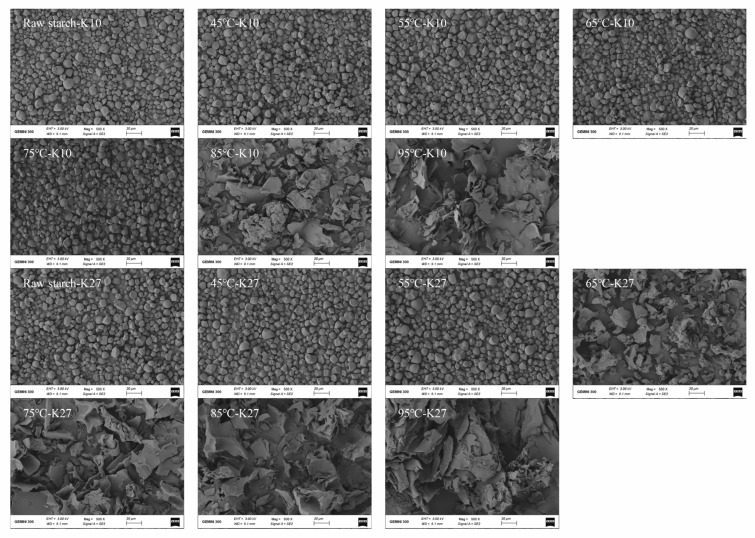
SEM images of kudzu starches with different DG prepared at 45, 55, 65, 75, 85, and 95 °C.

SEM was used to observe the morphology of kudzu starch granules (Figure 4). Kudzu starch granules were small, with an oval or polygonal shape, smooth surfaces, and no visible cracks. The morphological characteristics of *Pueraria thomsonii* starch (K10) and *Pueraria lobate* starch (K27) were relatively similar, making it challenging to distinguish obvious differences initially.

As the treatment temperature increased, the DG of the starches increased. This caused the granules to stick together, become irregular in shape, and gradually rupture. These changes confirmed the occurrence of gelatinization [35,38]. Specifically, after K10 was treated at 65 °C, its starch granules showed notable changes: the surface transformed from being smooth to rough. With further increases in temperature, the granules became increasingly fragmented, and their structure completely disappeared after the treatment at 85 °C. In contrast, K27 exhibited significant morphological changes at lower treatment temperatures. After treatment at 55 °C, its granules were fragmented but retained part of their structure, which completely disappeared after the treatment at 65 °C. These observations confirmed the characterization of gelatinization behavior and highlighted that different varieties of kudzu starch exhibited varying sensitivities to temperature during gelatinization. These differences may be attributed to the intrinsic structure and chemical composition of their granules [64,65]. Thus, for different varieties of kudzu starch, the DG—rather than the treatment temperature—is the key factor driving significant changes in granule morphology.

### 3.6. Effects of Different DG on the Long-Range Ordered Structure of Kudzu Starch

**Figure 5:**
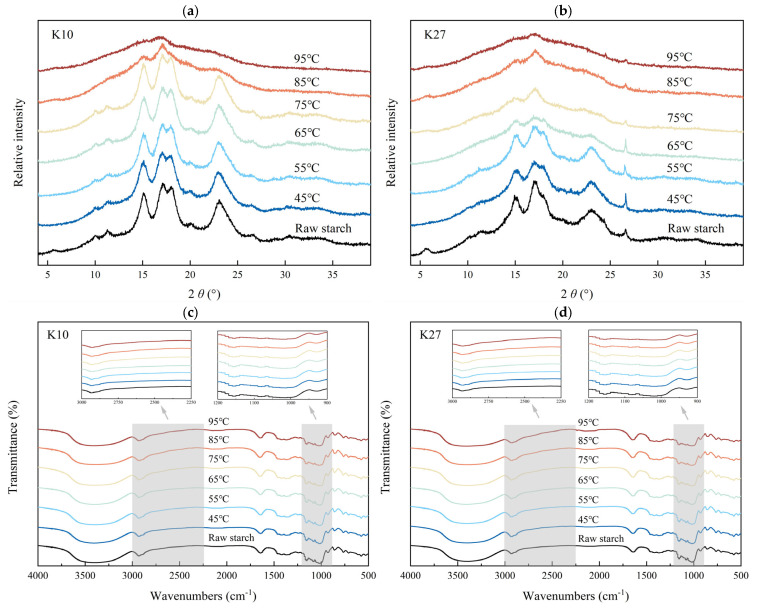
Ordered structure analysis of kudzu starches with different DG. (**a**) XRD of K10; (**b**) XRD of K27; (**c**) FTIR of K10; (**d**) FTIR of K27.

The semi-crystalline type and relative crystallinity (RC) of the starch samples were analyzed using X-ray diffractometry (XRD) (Figure 5 and Table S3). The XRD patterns of the two kudzu starches displayed strong peaks at 15.3°, 17–18° (doublet), and 23°, and weak peaks at 6°, 10–12° (doublet), and 26° (2*θ*). These features indicated a typical C-type crystallinity pattern. Notably, the strong peaks of *Pueraria thomsonii* starch (K10) were sharper than those of *Pueraria lobate* starch (K27), while the weak peaks of K27 were clearer than those of K10. As the DG increased, the crystal structures of both kudzu starches gradually disappeared, with the crystalline region transitioning to an amorphous state and the loss of birefringence [66].

For *Pueraria thomsonii* starch (K10), when the treatment temperature was lower than 75 °C, its weak peak at 2*θ* = 6° disappeared and the RC increased. This may be attributed to the increase in the mobility of the starch chains and the stabilization of the helical structure due to the low-temperature hydrothermal treatment, which altered crystalline and amorphous regions of the starch granules [67]. However, when the temperature exceeded 75 °C, the RC decreased significantly. High temperatures led to swelling and rupture of starch granules, destroying the crystalline regions inside the granules [56]. Additionally, hydrogen bonds between starch molecules were disrupted, weakening intermolecular interactions and thus breaking down the crystalline structure [68]. High temperatures also led to deconvolution of the double helix structure, exacerbating the destruction of the crystalline regions [56]. Furthermore, starch molecular chains may fracture at elevated temperatures, reducing the ordered molecular arrangement [69]. These combined physical and chemical changes resulted in a marked decrease in the RC of the starches at high temperatures.

The progressive disruption of long-range order with increasing DG aligns with the general behavior of starches under thermal treatment. However, the specific response of C-type kudzu starch can be better understood by comparison with other polymorphic types. For instance, Chi et al. [70] reported that dry heating markedly decreased the RC of potato starch (B-type), whereas its effect on normal maize starch (A-type) was negligible. This suggests that B-type crystallites—and consequently the B-type fraction within C-type starches—are more susceptible to thermal degradation. Similarly, Zhang et al. [71] observed that the semi-crystalline lamellae of legume starches (C-type), such as pea and mung bean, were completely disrupted above 75 °C, consistent with the sharp RC decline observed in K10 and K27 at elevated temperatures (>75 °C) in this study. These comparisons indicate that the critical temperature for crystalline disintegration is not only determined by the polymorphic type but also by the botanical origin and the intrinsic stability of the crystalline domains.

In contrast, the temperature at which *Pueraria lobate* starch (K27) showed a sudden drop in RC was significantly lower than that for K10, accompanied by some fluctuation. This fluctuation may be due to the differences in the degree of granule swelling and rupture at different temperatures, the disruption and rearrangement of intermolecular hydrogen bonds, and the degradation and reorganization of starch molecular chains [68,72].

### 3.7. Effects of Different DG on the Short-Range Ordered Structure of Kudzu Starch

Fourier Transform Infrared Spectroscopy (FTIR) was used to analyze the short-range ordered structure of kudzu starch with different DG. Gelatinization is a physical process that does not alter the chemical structure of starch [69]. The change in absorption wavelength of kudzu starch with different DG were minimal (Figure 5), indicating that the molecular structure of kudzu starch remained largely intact. No significant damage to the molecular structure or the emergence of new functional groups was observed. The observed changes were primarily associated with the breaking of hydrogen bonds within the molecule and the stretching of the molecular structure [4].

The two kudzu starches exhibited noticeable changes in the spectral region from 2250 to 2900 cm^−1^, corresponding to the -CH stretching vibration [4]. With the increase in treatment temperature, the spectral region, which was initially relatively flat, became more slope-like. During the gelatinization process, significant changes in hydrogen bonding within the starch molecules occurred. As the temperature increased, the hydrogen bonds within the starch granules were disrupted, resulting in a decrease in the ordered arrangement of the starch molecular chains and an increase in the amorphous regions. This disruption was irreversible, causing the starch granules to swell and eventually disintegrate into a homogeneous pasty solution [34,35].

Although the breaking of hydrogen bonds is the primary event during the gelatinization process, the rearrangement of starch molecular chains can lead to the formation of new hydrogen bonds. These new bonds may form within or between unpasted starch molecules and other pasted starch molecules, influencing the texture and stability of the final paste. However, these newly formed hydrogen bonds are typically less stable than those in the original crystalline structure [7,35]. For *Pueraria thomsonii* starch (K10), this change primarily occurred at the treatment temperatures above 65 °C, whereas for *Pueraria lobata* starch (K27), it occurred at above 55 °C.

A more detailed interpretation of the short-range order was obtained from the ratio of absorbance at 1047 cm^−1^ to 1022 cm^−1^ (R_1047/1022_). This ratio specifically reflects the proportion of ordered double helices to amorphous regions [73]. The evolution of R_1047/1022_ in relation to DG and RC reveals a critical sequence of structural disassembly. For both kudzu starches, R_1047/1022_ values showed only minor fluctuations at lower temperatures (45–75 °C) and low DG, indicating that the short-range double-helical order was largely preserved. This initial stability suggests that the early stage of heating primarily affects the long-range packing of helices into crystallites—as reflected by the initial changes in RC—while the helical conformation itself remains intact.

A pronounced decrease in R_1047/1022_ occurred between 75 °C and 95 °C, coinciding with the major loss of RC and high DG values. These findings demonstrate that increasing DG drives a coordinated disassembly across multiple structural levels, ultimately leading to a pronounced disruption of ordered crystalline regions, as reflected by the substantial decline in R_1047/1022_ [74]. Moreover, this sequential disassembly—where the loss of long-range crystallinity is accompanied by and preceded by the breakdown of short-range helical order—has also been reported by Zhang et al. [71] in other legume starches during gelatinization.

### 3.8. Effects of Different DG on the Apparent Viscosity of Kudzu Starch

The flow behavior of fully gelatinized kudzu starch pastes was fitted using the power-law model (Table S2), which provided an excellent fit with R^2^ values above 0.99. The flow behavior index (n) for all samples was significantly less than 1, confirming the typical shear-thinning behavior of pseudoplastic fluids [75]. K10 exhibited a lower n value compared to K27, while the consistency coefficient (*K*) showed no significant difference between them. The lower n value of K10 indicates a more pronounced shear-thinning behavior, suggesting that its internal structure was more susceptible to disruption under applied shear.

**Figure 6:**
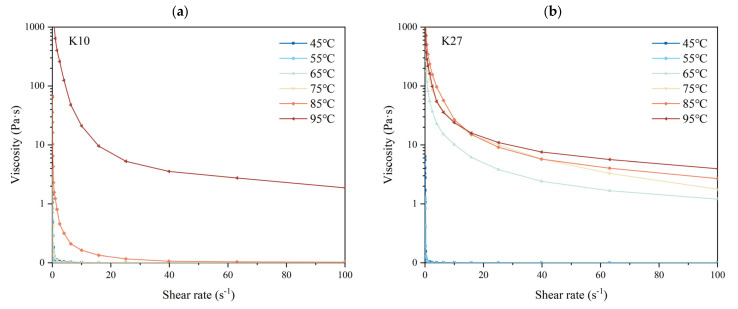
Apparent viscosity of kudzu starches with different DG. (**a**) K10; (**b**) K27.

This shear-thinning behavior, also observed as a decrease in viscosity with increasing shear rate (Figure 6), was primarily attributed to interactions among polymer colloidal particles [76]. At rest or under low shear rates, the molecular chains were highly entangled. As the shear rate increased, these entanglements were disrupted, and the destruction of intermolecular bonds outpaced their re-formation, thereby reducing the resistance to flow and leading to a decline in viscosity [77].

The viscosity of kudzu starch paste at the same shear rate was positively correlated with processing temperature. *Pueraria thomsonii* starch (K10) showed a marked viscosity increase above 85 °C, whereas *Pueraria lobata* starch (K27) exhibited a significant increase from 65 °C onwards. These results were consistent with the DG findings. This rise in viscosity is likely linked to amylose release and the extent of granule disintegration. As the DG increased, more starch granules swelled and ruptured, releasing a greater amount of molecular chains, especially amylose, into the solution. This led to greater molecular entanglements within the fluid, which increased the internal resistance of the fluid, thereby raising its viscosity [6,34]. Additionally, a higher DG indicated that more starch molecules contributed to the formation of a colloidal network structure, which was significantly reinforced by the leached amylose, enhancing the resistance to deformation under shear forces, further increasing the viscosity of the fluid [76]. Therefore, the positive correlation between DG and paste viscosity can be explained by more complete granule disintegration and the consequent release and interaction of starch polymers, particularly amylose.

### 3.9. DG as a Key Determinant of the Multifunctional Properties of Kudzu Starch

**Figure 7:**
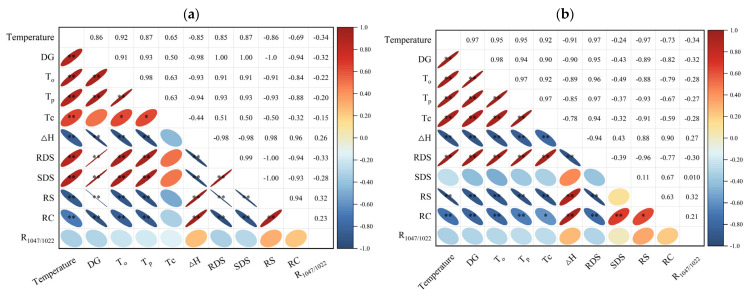
Correlation analysis of kudzu starches with different DG. (**a**) K10; (**b**) K27. Statistical significance is denoted as * *p* < 0.05, ** *p* < 0.01.

The treatment temperature exhibited a highly significant positive correlation with the DG of kudzu starch (*p* < 0.01) (Figure 7). More importantly, DG served as a pivotal linker that systematically coordinated the structural, thermal, and digestive properties of the starch, which varied among different varieties. For *Pueraria thomsonii* starch (K10), the DG exhibited highly significant positive correlations (*p* < 0.01) with *T*_o_, *T*_p_, RDS content and SDS content; highly significant negative correlations (*p* < 0.01) with ∆*H*, RS content, and RC. For *Pueraria lobate* starch (K27), the DG showed highly significant positive correlation (*p* < 0.01) with *T*_o_, *T*_p_, *T*_c_ and RDS content; and highly significant negative correlation (*p* < 0.01) with ∆*H*, RS content, and RC.

The results indicated that DG can be adjusted by modifying processing conditions, which directly influence the functional properties of starch. By increasing DG, the gelatinization temperatures (*T*_o_, *T*_p_, *T*_c_) of kudzu starch increased, thereby enhancing its thermal stability. The starch with higher pasting temperature can provide better processing performance and product quality for foods requiring high temperature processing, and is expected to be applied in the production of canned foods, pastas and baked foods [65]. Moreover, increasing DG can also disrupt the crystal structure of starch granules and alter their physicochemical properties, which in turn impacts the digestive properties of starch, increases the RDS content and decreases the RS content [12]. This modified starch, with its tunable digestibility, is expected to be used as a key ingredient in formulated foods for special medical purposes (FSMPs) and nutritional formulations for the elderly, which can help individuals with gastrointestinal dysfunction to achieve rapid digestion and absorption, enhance energy provision, and mitigate gastrointestinal stress [78,79].

## 4. Conclusions

This study demonstrates that the dextrinization degree (DG) of kudzu starch, precisely controllable via hydrothermal temperature, serves as the primary driver for tailoring its physicochemical, structural, and digestive properties, with cultivar-specific modulation observed. The strong correlations link increased DG to higher gelatinization temperatures and digestibility but lower gelatinization enthalpy, resistant starch content, and crystallinity. These findings provide a practical basis for designing functional foods, such as low-glycemic options from low-DG starch or instant products from high-DG starch. While this approach is promising, future work must address limitations including retrogradation behavior, paste stability, and reproducibility under industrial conditions to assess commercial viability.

## Figures and Tables

**Table 1:** Effects of different DG on thermal properties of kudzu starch.

Samples	*T*_o_ (°C)	*T*_p_ (°C)	*T*_c_ (°C)	∆*H* (J/g)
K10				
45 °C	79.85 ± 0.39 ^d^	84.85 ± 0.39 ^c^	89.05 ± 2.61 ^b^	17.64 ± 0.38 ^d^
55 °C	80.42 ± 0.45 ^cd^	85.53 ± 0.35 ^c^	91.52 ± 0.56 ^ab^	16.91 ± 0.48 ^c^
65 °C	80.50 ± 0.30 ^c^	85.09 ± 0.42 ^c^	90.66 ± 0.74 ^ab^	15.58 ± 0.68 ^b^
75 °C	83.25 ± 0.20 ^b^	86.85±0.16 ^b^	92.65 ± 1.38 ^a^	13.80 ± 0.81 ^a^
85 °C	85.69 ± 0.22 ^a^	89.18 ± 0.54 ^a^	92.48 ± 0.22 ^a^	2.79 ± 0.13 ^a^
95 °C	-	-	-	-
K27				
45 °C	61.08 ± 0.30 ^d^	66.52 ± 2.35 ^d^	76.62 ± 1.04 ^d^	13.26 ± 0.70 ^d^
55 °C	58.67 ± 0.28 ^d^	66.76 ± 2.28 ^d^	76.78 ± 0.65 ^d^	11.97 ± 0.85 ^cd^
65 °C	72.48 ± 0.05 ^c^	76.77 ± 0.46 ^c^	81.47 ± 2.94 ^c^	2.55 ± 0.06 ^c^
75 °C	79.93 ± 0.83 ^b^	95.63 ± 0.91 ^b^	102.06 ± 3.79 ^b^	2.10 ± 0.11 ^b^
85 °C	90.96 ± 4.12 ^a^	102.07 ± 3.93 ^a^	107.59 ± 1.77 ^a^	1.35 ± 0.18 ^a^
95 °C	-	-	-	-

Note: Data are presented as means ± standard deviations, with different letters in the same column indicating significant differences (*p* < 0.05). *T*_o_, onset temperature; *T*_p_, peak temperature; *T*_c_, conclusion temperature; ∆*H*: gelatinization enthalpy.

## Data Availability

The original contributions presented in this study are included in the article/Supplementary Materials. Further inquiries can be directed to the corresponding author.

## Supplementary Materials

The following supporting information can be downloaded at: https://www.mdpi.com/article/10.3390/foods14213614/s1, Figure S1: Three different methods for measuring the DG of kudzu starch: (**a**) Enzymatic method; (**b**) DSC method; (**c**) Iodine-binding method; Table S1: Characteristics of enzymatic, DSC and iodine-binding methods; Table S2: Particle size distribution, amylose content, amylose leaching (95 °C), digestive, thermal and rheological parameters of kudzu starch; Table S3: RC and R_1047/1022_ of kudzu starch.

## References

[1] Li M., Miao M., Sun J., Fang H., Liu L., Xu X., Zheng Y., Lai Q., Tang Y., Liu X. (2024). Structure and physicochemical properties of starches from six accessions of the genus *Pueraria* in China. Int. J. Biol. Macromol..

[2] Zhu B., Huang F., Guo J., Song K., He J., Liu S., Zhou X. (2024). Unveiling the hidden potential of *Pueraria lobata*: A comprehensive analysis based on fiber morphology and physicochemical properties. J. Mater. Sci..

[3] Zhao Y., Zhu X., Fang Y. (2021). Structure, properties and applications of kudzu starch. Food Hydrocoll..

[4] He R., Li M., Huang B., Zou X., Li S., Sang X., Yang L. (2024). Comparative analysis of multi-angle structural alterations and cold-water solubility of kudzu starch modifications using different methods. Int. J. Biol. Macromol..

[5] Niu Z., Li M., Hou X., Qiao D., Cheng Z., Zhang L., Zhang B. (2023). Shortening growth year improves functional features of kudzu starch by tailoring its multi-scale structure. Int. J. Biol. Macromol..

[6] Schirmer M., Jekle M., Becker T. (2015). Starch gelatinization and its complexity for analysis. Starch-Stärke.

[7] Donmez D., Pinho L., Patel B., Desam P., Campanella O.H. (2021). Characterization of starch-water interactions and their effects on two key functional properties: Starch gelatinization and retrogradation. Curr. Opin. Food Sci..

[8] Jiang J., Li J., Han W., Yang Q., Liu Q., Xiao H., Lin Q., Fang Y. (2022). Effects of reheating methods on rheological and textural characteristics of rice starch with different gelatinization degrees. Foods.

[9] Ji L., Zhang H., Cornacchia L., Sala G., Scholten E. (2022). Effect of gelatinization and swelling degree on the lubrication behavior of starch suspensions. Carbohydr. Polym..

[10] Parada J., Aguilera J.M. (2009). In vitro digestibility and glycemic response of potato starch is related to granule size and degree of gelatinization. J. Food Sci..

[11] Li S., Wang Z., Feng D., Pan Y., Li E., Wang J., Li C. (2024). The important role of starch fine molecular structures in starch gelatinization property with addition of sugars/sugar alcohols. Carbohydr. Polym..

[12] Yan X., McClements D.J., Luo S., Liu C., Ye J. (2024). Recent advances in the impact of gelatinization degree on starch: Structure, properties and applications. Carbohydr. Polym..

[13] Lacerda L.D., da Silveira N.P., Bondam A.F., Hoffmann J.F. (2024). Starch gelatinization behavior: The impact of granular structure. Starch-Stärke.

[14] Gandhi N., Singh B., Singh P., Sharma S. (2021). Functional, rheological, morphological, and micro-structural properties of extrusion-processed corn and potato starches. Starch-Stärke.

[15] Li S., Dong S., Fang G., Hao Y., Gao Q. (2022). Study on internal structure and digestibility of jackfruit seed starch revealed by chemical surface gelatinization. Food Hydrocoll..

[16] Liu K., Liu Q. (2020). Enzymatic determination of total starch and degree of starch gelatinization in various products. Food Hydrocoll..

[17] Yang Y., Li M., Wang Q., Huang H., Zhao Y., Du F., Chen Y., Shen J., Luo H., Zhao Q. (2022). Pueraria lobata starch regulates gut microbiota and alleviates high-fat high-cholesterol diet induced non-alcoholic fatty liver disease in mice. Food Res. Int..

[18] Kong X., Zhu P., Sui Z., Bao J. (2015). Physicochemical properties of starches from diverse rice cultivars varying in apparent amylose content and gelatinisation temperature combinations. Food Chem..

[19] Gunaratne A., Hoover R. (2002). Effect of heat-moisture treatment on the structure and physicochemical properties of tuber and root starches. Carbohydr. Polym..

[20] Zuo Y., Zhu F., Jiang S., Sui Z., Kong X. (2024). Structural, physicochemical, and digestive properties of starch-tannic acid complexes modulated by co-heating temperatures. Food Hydrocoll..

[21] Liu Q., Wang Y., Yang Y., Yu X., Xu L., Jiao A., Jin Z. (2023). Structure, physicochemical properties and in vitro digestibility of extruded starch-lauric acid complexes with different amylose contents. Food Hydrocoll..

[22] Tian J., Chen S., Wu C., Chen J., Du X., Chen J., Liu D., Ye X. (2016). Effects of preparation methods on potato microstructure and digestibility: An in vitro study. Food Chem..

[23] Goni I., GarciaAlonso A., SauraCalixto F. (1997). A starch hydrolysis procedure to estimate glycemic index. Nutr. Res..

[24] Takahama U., Hirota S. (2010). Fatty Acids, Epicatechin-dimethylgallate, and rutin interact with buckwheat starch inhibiting its digestion by amylase: Implications for the decrease in glycemic index by buckwheat flour. J. Agric. Food Chem..

[25] Moraes J., Branzani R.S., Franco C.M.L. (2014). Behavior of Peruvian carrot (*Arracacia xanthorrhiza*) and cassava (*Manihot esculenta*) starches subjected to heat-moisture treatment. Starch-Stärke.

[26] Xiao Y., Shen M., Luo Y., Ren Y., Han X., Xie J. (2020). Effect of *Mesona chinensis* polysaccharide on the pasting, rheological, and structural properties of tapioca starch varying in gelatinization temperatures. Int. J. Biol. Macromol..

[27] Oskaybas-Emlek B., Ozbey A., Aydemir L.Y., Kahraman K. (2022). Production of buckwheat starch-myristic acid complexes and effect of reaction conditions on the physicochemical properties, X-ray pattern and FT-IR spectra. Int. J. Biol. Macromol..

[28] Chen X., He X., Zhang B., Fu X., Li L., Huang Q. (2018). Structure, physicochemical and in vitro digestion properties of ternary blends containing swollen maize starch, maize oil and zein protein. Food Hydrocoll..

[29] Sims I.M., Smith A.M., Morris G.A., Ghori M.U., Carnachan S.M. (2018). Structural and rheological studies of a polysaccharide mucilage from lacebark leaves (*Hoheria populnea* A. Cunn.). Int. J. Biol. Macromol..

[30] Wang S., Copeland L. (2013). Molecular disassembly of starch granules during gelatinization and its effect on starch digestibility: A review. Food Funct..

[31] Saito K., Okouchi M., Yamaguchi M., Takechi T., Hatanaka Y., Kitsuda K., Mannari T., Takamura H. (2022). Effect of the addition of high-temperature water on the properties of batter and bread made from gluten-free rice flour. J. Food Sci..

[32] Ohmura M., Matsumiya K., Tatsuro M., Fujita A., Hayashi Y., Matsumura Y. (2021). Change in surface structure and inner microstructure of durum wheat pasta during the boiling process. LWT-Food Sci. Technol..

[33] Biliaderis C.G. (1983). Differential scanning calorimetry in food research: A review. Food Chem..

[34] Wang S., Zhang X., Wang S., Copeland L. (2016). Changes of multi-scale structure during mimicked DSC heating reveal the nature of starch gelatinization. Sci. Rep..

[35] Wang Y., Chen L., Yang T., Ma Y., McClements D.J., Ren F., Tian Y., Jin Z. (2021). A review of structural transformations and properties changes in starch during thermal processing of foods. Food Hydrocoll..

[36] He R., Li S., Zhao G., Zhai L., Qin P., Yang L. (2023). Starch modification with molecular transformation, physicochemical characteristics, and industrial usability: A state-of-the-art review. Polymers.

[37] Pesek S., Lehene M., Branzanic A.M.V., Silaghi-Dumitrescu R. (2022). On the origin of the blue color in the iodine/iodide/starch supramolecular complex. Molecules.

[38] Wang S., Chao C., Xiang F., Zhang X., Wang S., Copeland L. (2018). New insights into gelatinization mechanisms of cereal endosperm starches. Sci. Rep..

[39] Naguleswaran S., Vasanthan T., Hoover R., Bressler D. (2014). Amylolysis of amylopectin and amylose isolated from wheat, triticale, corn and barley starches. Food Hydrocoll..

[40] Di Paola R.D., Asis R., Aldao M.A.J. (2003). Evaluation of the degree of starch gelatinization by a new enzymatic method. Starch-Stärke.

[41] Chi C., Li X., Huang S., Chen L., Zhang Y., Li L., Miao S. (2021). Basic principles in starch multi-scale structuration to mitigate digestibility: A review. Trends Food Sci. Technol..

[42] Gao K., Zha F., Yang Z., Rao J., Chen B. (2022). Structure characteristics and functionality of water-soluble fraction from high-intensity ultrasound treated pea protein isolate. Food Hydrocoll..

[43] Zhong Y., Liu L., Qu J., Blennow A., Hansen A.R., Wu Y., Guo D., Liu X. (2020). Amylose content and specific fine structures affect lamellar structure and digestibility of maize starches. Food Hydrocoll..

[44] Butterworth P.J., Warren F.J., Grassby T., Patel H., Ellis P.R. (2012). Analysis of starch amylolysis using plots for first-order kinetics. Carbohydr. Polym..

[45] Corgneau M., Gaiani C., Petit J., Nikolova Y., Banon S., Ritie-Pertusa L., Doan Thanh Lam L., Scher J. (2019). Digestibility of common native starches with reference to starch granule size, shape and surface features towards guidelines for starch-containing food products. Int. J. Food Sci. Technol..

[46] Pham Van H., Huynh Thi C., Nguyen Thi Lan P. (2016). In vitro digestibility and in vivo glucose response of native and physically modified rice starches varying amylose contents. Food Chem..

[47] Romano A., Mackie A., Farina F., Aponte M., Sarghini F., Masi P. (2016). Characterisation, in vitro digestibility and expected glycemic index of commercial starches as uncooked ingredients. J. Food Sci. Technol..

[48] Chisbert M., Castell A.-L., Vinoy S., Nazare J.-A. (2024). The impact of slowly digestible and resistant starch on glucose homeostasis and insulin resistance. Curr. Opin. Clin. Nutr. Metab. Care.

[49] Guo J., Tan L., Kong L. (2022). Multiple levels of health benefits from resistant starch. J. Agric. Food Res..

[50] Shen L., Li J., Li Y. (2022). Resistant starch formation in rice: Genetic regulation and beyond. Plant Commun..

[51] Tamura M., Fujimoto A., Kitamura R., Saito T., Mikami A., Susaki K., Kobayashi H. (2025). Structural characteristics determining starch digestibility in cooked rice complexed with an emulsion formulation. Food Chem..

[52] Atkinson F.S., Brand-Miller J.C., Foster-Powell K., Buyken A.E., Goletzke J. (2021). International tables of glycemic index and glycemic load values 2021: A systematic review. Am. J. Clin. Nutr..

[53] Yu W., Zhou X., Li C. (2021). Application of first-order kinetics modeling to reveal the nature of starch digestion characteristics. Food Funct..

[54] Ma S., Zuo J., Chen B., Fu Z., Lin X., Wu J., Zheng B., Lu X. (2024). Structural, properties and digestion in vitro changes of starch subjected to high pressure homogenization: An update review. Int. J. Biol. Macromol..

[55] Vamadevan V., Bertoft E. (2020). Observations on the impact of amylopectin and amylose structure on the swelling of starch granules. Food Hydrocoll..

[56] Wang B., Gao W., Kang X., Dong Y., Liu P., Yan S., Yu B., Guo L., Cui B., Abd El-Aty A.M. (2021). Structural changes in corn starch granules treated at different temperatures. Food Hydrocoll..

[57] Qiao J., Jia M., Niu J., Zhang Z., Xing B., Liang Y., Li H., Zhang Y., Ren G., Qin P. (2024). Amylopectin chain length distributions and amylose content are determinants of viscoelasticity and digestibility differences in mung bean starch and proso millet starch. Int. J. Biol. Macromol..

[58] Vamadevan V., Bertoft E., Soldatov D.V., Seetharaman K. (2013). Impact on molecular organization of amylopectin in starch granules upon annealing. Carbohydr. Polym..

[59] Hickman B.E., Janaswamy S., Yao Y. (2009). Properties of starch subjected to partial gelatinization and β-amylolysis. J. Agric. Food Chem..

[60] Qi W., Kong S., Li X., Peng Z., Sun L., Wang Z., Cheng J. (2024). Insight into characteristics in rice starch under heat- moisture treatment: Focus on the structure of amylose/amylopectin. Food Chem. X.

[61] Arns B., Bartz J., Radunz M., do Evangelho J.A., Pinto V.Z., Zavareze E.d.R., Guerra Dias A.R. (2015). Impact of heat-moisture treatment on rice starch, applied directly in grain paddy rice or in isolated starch. LWT-Food Sci. Technol..

[62] Bertoft E., Annor G., Vamadevan V., Lin A.H.-M. (2025). On the architecture of starch granules revealed by iodine binding and lintnerization. Part 2: Molecular structure of lintnerized starches. Biopolymers.

[63] Pesek S., Silaghi-Dumitrescu R. (2024). The iodine/iodide/starch supramolecular complex. Molecules.

[64] Li C. (2022). Recent progress in understanding starch gelatinization-An important property determining food quality. Carbohydr. Polym..

[65] Chakraborty I., Pooja N., Mal S.S., Paul U.C., Rahman M.H., Mazumder N. (2022). An insight into the gelatinization properties influencing the modified starches used in food industry: A review. Food Bioprocess Technol..

[66] Gou M., Wu H., Saleh A.S.M., Jing L., Liu Y., Zhao K., Su C., Zhang B., Jiang H., Li W. (2019). Effects of repeated and continuous dry heat treatments on properties of sweet potato starch. Int. J. Biol. Macromol..

[67] Zhang S., Wang Z., Zhou X., Song Y., Wang L., Tian H., Zhang D., Lue X., Liu F., Huang J. (2025). Insights into the regulation mechanisms of dual hydrothermal treatment on the structure and digestive characteristics of A- and B-type wheat starch granules. Food Res. Int..

[68] Huang X., Liu H., Ma Y., Mai S., Li C. (2022). Effects of extrusion on starch molecular degradation, order-disorder structural transition and digestibility: A review. Foods.

[69] Liu X., Huang S., Chao C., Yu J., Copeland L., Wang S. (2022). Changes of starch during thermal processing of foods: Current status and future directions. Trends Food Sci. Technol..

[70] Chi C., Li X., Lu P., Miao S., Zhang Y., Chen L. (2019). Dry heating and annealing treatment synergistically modulate starch structure and digestibility. Int. J. Biol. Macromol..

[71] Zhang X., Zhu C., Geng D., Cheng Y., Tang N. (2025). Characterization of dynamic of the structural changes of legume starches during gelatinization. Int. J. Biol. Macromol..

[72] Chen Z., Huang J., Pu H., Keipper W. (2022). The effects of temperature on starch molecular conformation and hydrogen bonding. Starch-Stärke.

[73] Wang S., Wang J., Zhang W., Li C., Yu J., Wang S. (2015). Molecular order and functional properties of starches from three waxy wheat varieties grown in China. Food Chem..

[74] An H., Ma Q., Zhang F., Zhai C., Sun J., Tang Y., Wang W. (2024). Insight into microstructure evolution during starch retrogradation by infrared and Raman spectroscopy combined with two-dimensional correlation spectroscopy analysis. Food Hydrocoll..

[75] Yang K., Luo X., Zhai Y., Liu J., Chen K., Shao X., Wu X., Li Y., Chen Z. (2021). Influence of sodium alginate on the gelatinization, rheological, and retrogradation properties of rice starch. Int. J. Biol. Macromol..

[76] Duan X., Liu Q., Zhao R., Liu W., Zhang L., Hu H. (2024). Effects of particle properties, intermolecular forces, and molecular structure on the shear-thickening behavior of waxy starch dispersions. Carbohydr. Polym..

[77] Dimonie D., Grigorescu R.-M., Trica B., Raduly M., Damian C.-M., Trusca R., Mustatea A.-E., Dima S.-O., Oancea F. (2024). De- and re-structuring of starch to control the melt and solid state visco-elasticity as method for getting new multi component compounds with scalable properties. Polymers.

[78] Carpentieri S., Larrea-Wachtendorff D., Barbosa-Canovas G.V., Ferrari G. (2024). In vitro digestibility of rice and tapioca starch-based hydrogels produced by high-pressure processing (HPP). Innov. Food Sci. Emerg. Technol..

[79] Satusap P., Chavasit V., Kriengsinyos W., Judprasong K. (2014). Development of cereal and legume based food products for the elderly. Springerplus.

