# Supplementary material for: Effects of Different Degrees of Gelatinization on Structural, Physicochemical and Digestive Properties of Kudzu Starch
Source: Foods. 2025 Oct 23;14(21):3614. doi: 10.3390/foods14213614 (PMC12607623; doi:10.3390/foods14213614)
Supplement: Supplementary file 1 [file foods-14-03614-s001.zip › foods-3925787-supplementary.pdf]

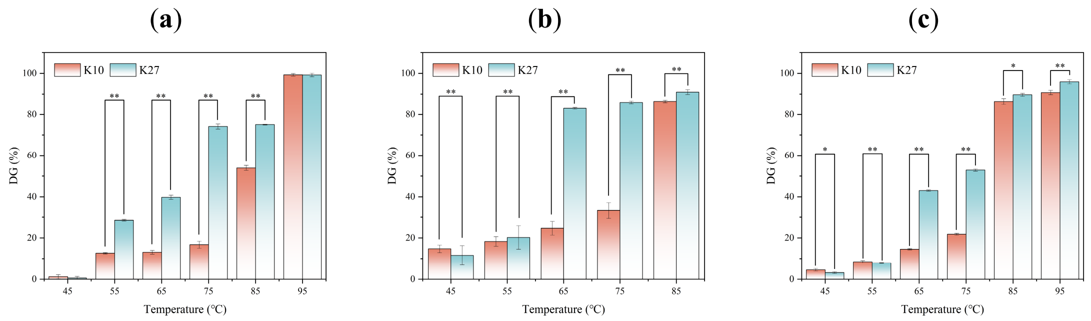

Figure S1. Three different methods for measuring the DG of kudzu starch: (**a**) Enzymatic method; (**b**) DSC method; and (**c**) Iodine-binding method. Statistical analysis was performed using Student's t-test (* $p < 0.05$, ** $p < 0.01$).

Table S1. Characteristics of enzymatic, DSC and iodine-binding methods.

Methods	Principle	Advantages	Disadvantages	Main influencing factors
Enzymatic method	the linear relationship between DG and the enzymatic sensitivity of starch	the effects of different temperature treatments on DG can be more fully reflected	high costs and complex conditions	starch granule size and morphology
DSC method	Enthalpy reduction of starch after gelatinization	easy operation	large errors and limited temperature range of measurement	double helix structure, crystal structure of starch
Iodine-bonding method	increased leaching of amylose after gelatinization	relatively easy to perform, accuracy intermediate	not suitable for temperatures above 85 °C	leaching of amylose

	and its ability to bind iodine	between enzymatic and DSC methods

Table S2. Particle size distribution, amylose content, amylose leaching (95 ℃), digestive, thermal and rheological parameters of kudzu starch.

Properties	K10	K27
D_{10} (μm)	2.76±0.46^{b}	5.31±0.39^{a}
D_{50} (μm)	8.76±0.36^{b}	9.55±0.22^{a}
D_{90} (μm)	14.15±0.29^{b}	15.08±0.14^{a}
Span	1.30±0.13^{b}	1.03±0.07^{a}
$D_{[4,3]}$ (μm)	8.99±0.30^{a}	9.63±0.25^{a}
Amylose content (%)	27.69±0.22^{a}	26.51±0.46^{b}
Amylose leaching (95 ℃,%)	3.17±0.17^{a}	2.98±0.06^{a}
RDS (%)	78.21±0.82^{b}	81.18±0.56^{a}
SDS (%)	7.38±1.22^{b}	12.69±0.54^{a}
RS (%)	14.41±0.47^{a}	6.13±0.68^{b}
k ($\times 10^{-2}$ min^{-1})	15.93±2.27^{a}	12.16±0.21^{b}
C_{∞} (%)	81.21±0.62^{b}	86.48±0.43^{a}
AUC	8.74±1.11^{a}	7.22±0.12^{b}
HI	7.28±0.93^{a}	6.02±0.10^{b}
eGI	43.71±0.51^{a}	43.01±0.06^{b}
T_o (℃)	80.36±0.09^{a}	61.72±0.16^{b}
T_p (℃)	85.35±0.41^{a}	67.42±0.05^{b}
T_c (℃)	96.97±2.17^{a}	77.20±0.65^{b}
ΔH (J/g)	20.68±2.57^{a}	24.54±1.02^{a}
n	0.081±0.164^{b}	0.120±0.008^{a}
K ($Pa \cdot s^{n}$)	1.655±0.441^{a}	1.414±0.049^{a}
R^2	0.997	0.999

Note: Data are presented as means ± standard deviations, with different letters in the same row indicating significant differences (p < 0.05). D_{10}, 10th percentile particle size; D_{50}, median particle size; D_{90}, 90th percentile particle size; Span, particle size distribution span; $D_{[4,3]}$, volume-weighted mean particle size; RDS, rapidly digestible starch content; SDS, slowly digestible starch content; RS, resistant starch content; k, rate constant in digestion kinetics; C_∞, starch digestibility at the end of digestion; AUC, area under the hydrolysis curve; HI, hydrolysis index; eGI, estimated glycemic index; T_o, onset temperature; T_p, peak temperature; T_c, conclusion temperature; ΔH: gelatinization enthalpy; n, flow behavior index; K: consistency coefficient; R^2: determination coefficient.

Table S3. RC and $R_{1047/1022}$ of kudzu starch.

Samples	RC (%)	$R_{1047/1022}$
K10		
raw starch	15.36±0.27^{c}	0.913±0.019^{a}
45 °C	22.82±0.15^{b}	0.936±0.045^{a}
55 °C	24.06±0.41^{a}	0.841±0.034^{b}
65 °C	24.34±0.18^{a}	0.832±0.010^{b}
75 °C	23.29±0.30^{b}	0.849±0.019^{b}
85 °C	7.30±0.27^{d}	0.846±0.034^{b}
95 °C	5.24±0.13^{e}	0.831±0.027^{b}
K27		
raw starch	17.15±0.18^{a}	0.939±0.023^{a}
45 °C	12.17±0.17^{c}	0.901±0.028^{ab}
55 °C	14.06±0.74^{b}	0.883±0.037^{ab}
65 °C	4.66±0.48^{e}	0.881±0.016^{ab}
75 °C	8.38±0.29^{d}	0.882±0.043^{ab}
85 °C	5.39±0.43^{e}	0.870±0.022^{b}
95 °C	1.66±0.25^{f}	0.793±0.038^{c}

Note: Data are presented as means ± standard deviations, with different letters in the

same column indicating significant differences ($p < 0.05$). RC, relative crystallinity; $R_{1047/1022}$, the absorbance ratio of 1047 cm^{-1}/1022cm^{-1}.
